# Plastic surgery professional misconduct: a cross-sectional study on cases between 2008 and 2017, filed before the São Paulo State Medical Board

**DOI:** 10.1590/1516-3180.2020.0747.R1.22042021

**Published:** 2021-10-20

**Authors:** Paulo Cézar Mariani, Clóvis Francisco Constantino, Rui Nunes

**Affiliations:** I MD. Doctoral Student, Faculdade de Medicina da Universidade do Porto, Porto, Portugal.; II PhD. Physician and Professor, Universidade de Santo Amaro (UNISA), São Paulo (SP), Brazil.; III PhD. Professor, Faculdade de Medicina da Universidade do Porto, Porto, Portugal.

**Keywords:** Process assessments, health care, Ethics, medical, Surgery, plastic, Professional-misconduct cases, Medical ethics, Medical board investigations

## Abstract

**BACKGROUND:**

In plastic surgery, a lack of ethical and moral behavior by professionals can result in unfortunate circumstances and can justify ethical-disciplinary procedures.

**OBJECTIVE:**

To review 421 plastic surgery professional-misconduct cases filed before the São Paulo State Medical Board (CREMESP) between 2008 and 2017.

**DESIGN AND SETTING:**

Cross-sectional study conducted in a medical council.

**METHODS:**

The cases were categorized according to sex, age, medical specialty (plastic surgery, other field or none), medical ethics code chapter(s) involved, ethics code articles violated and board ruling/outcome.

**RESULTS:**

Most of the defendants were men over 40 years of age who were experienced in their professional practice and who graduated from public and private universities all over Brazil; 47.74% had a specialist title in plastic surgery. Violation of professional responsibility (medical malpractice, recklessness or negligence) was the commonest complaint (28.43%), followed by medical advertising (24.19%) and poor doctor-patient relationship (10.39%), in violation of articles 18, 51, 75 and 1. Among the 233 cases adjudicated over this period, 133 resulted in disciplinary sanction, 80 were ruled in the physician’s favor and 20 were dismissed.

**CONCLUSION:**

Classification of plastic surgery professional-misconduct cases creates possibilities for adopting preventive measures for good practice in this specialty, which would consequently reduce the number of complaints to the regional medical boards.

## INTRODUCTION

Plastic surgery is a medical procedure that consists of various surgical or non-surgical procedures that reshape human body parts. The purpose is to treat “anatomical, congenital, acquired, post-traumatic, degenerative and oncological deformities to improve patients’ biopsychosocial health and subsequently their quality of life”.^1^

Borges^1^ reported that there had been an explosive increase in lawsuits involving plastic-surgery malpractice, which has led to challenges for physicians and medical associations. Two phenomena explain this considerable increase in lawsuits: on the one hand, patients are increasingly more aware of their rights enshrined in articles 196 and 200 of the Brazilian Constitution, including redress for medical malpractice; and on the other hand, patients are clearly being affected by job instability, especially in the government, and by media influence, deteriorating doctor-patient relationships and inefficient university residency and postgraduate programs. These issues have led to greater legislative protections for patients seeking answers for care or treatment that was deemed unsatisfactory.^2^

Physicians commit ethical violations when they fail to follow medical guidelines.^3^^,^^4^^,^^5^^,^^6^ Therefore, civil liability for medical malpractice should be examined from two separate angles: a) liability for services directly and personally rendered by physicians as self-employed individuals; b) medical liability for rendering medical services as a business, which includes inpatient and outpatient hospitals, clinics, blood banks and medical laboratories.^7^^,^^8^

In medical practice, malpractice is understood as a wrongful act that damages or jeopardizes the health of another person and occasions poor or adverse result(s) owing to a physician’s action or inaction, in violation of medical guidelines.^9^^,^^10^^,^^11^ Malpractice can be qualified by any of the three types listed in article 1 of the medical ethics code, based on the Brazilian penal code, the Brazilian constitution or the Brazilian civil procedure code, namely negligence, recklessness and professional malpractice.

The article governing professional responsibility is the article most often violated by doctors.^12^ The Brazilian Medical Association (BMA) and regional medical boards are tasked with being notified of, investigating and ruling on all complaints filed, as well as for regulating the practice of medicine.^13^^,^^14^^,^^15^ Once a professional-misconduct case has been instituted, and once it has been established that there has been a violation of the medical ethics code, any of the five disciplinary sanctions listed in article 22 of Brazilian law 3.268/1957 can be handed down to the physician: confidential warning - classified notice (punishment A); confidential reprimand - classified notice (punishment B); public reprimand - notice on public record (punishment C); suspension of license to practice for up to 30 days (punishment D); or revocation of license to practice pending final ruling by the Brazilian Medical Association (punishment E). Either party may appeal against any punishment administered.^16^^,^^17^

## OBJECTIVE

The objective of this study was to classify cases relating to plastic surgery professional misconduct filed before the São Paulo State Medical Board (CREMESP) between 2008 and 2017, considering the demographic and professional characteristics: sex, age, private or public university education, medical specialty (plastic surgery, other field or none); and the case characteristics: medical ethics code chapter involved in the case, ethics code article(s) violated and ruling/outcome.

## METHODS

This was a retrospective cross-sectional database study that included all professional-misconduct cases involving plastic surgery that were filed before CREMESP over a 10-year period from 2008 to 2017. A total of 421 such cases were investigated (and closed) by CREMESP over this period. Notably, among these 421 cases, there were physicians under investigation in more than one case, such that 273 physicians, with or without a specialty qualification in plastic surgery, were involved.

We analyzed complaints involving plastic surgery in the CREMESP database after firstly receiving authorization from CREMESP (through a letter from its board, without reference number, but dated September 12, 2017) and then receiving ethics approval from the research ethics committee of the university where this study was conducted (no. 2.338.983; dated October 19, 2017). The board furnished the research team only with complaints involving plastic surgery, and the parties to the complaints were not identified. This research was conducted in accordance with the ethical and care standards and set forth in the Declaration of Helsinki and Nuremberg Code (Brazilian Medical Association Ruling 1785/2006). Professional-misconduct cases that had been dismissed or that were converted to administrative proceedings were not included.

The following variables were extracted from the database: sex, age, possession of medical specialty title in plastic surgery, case subject matter, medical ethics code article(s) violated and ruling/outcome.

The quantitative research design was descriptive, in accordance with the nature of the variable. The results are presented in tables, as described below.

## RESULTS

A total of 7,789 professional-misconduct cases of all natures were filed before CREMESP during the period in question. Of these, 421 cases (5.40%) involved 273 physicians with or without a specialty qualification in plastic surgery. All regional-board cases filed or pending before the CREMESP between 2008 and 2017 were included in the present review.

At the time of this review, among the 421 professional-misconduct cases that met the inclusion criterion, 233 (55.35%) cases had been adjudicated. Of these 233 cases, 133 (57%) resulted in disciplinary sanction, 80 (34.5%) were ruled in the physician’s favor and 20 (8.5%) were dismissed. A total of 188 cases (44.65%) were still pending investigation and judgment at the end of the year 2017, when data were collected for this review.

### Classification of defendants

Table 1 lists the general characteristics of the defendant physicians: sex, age, type of university education and whether they had a medical specialty title in plastic surgery.

**Table 1.** Breakdown of the 421 cases according to sex, age, type of university education, and possession of a specialty title in plastic surgery, in numbers and percentages

Physician profile	n	%
Sex		
Male	325	77.19
Female	96	22.81
**Total**	**421**	**100.00**
Age (years)		
30-39	109	25.89
40-49	137	32.54
50-59	85	20.19
60-69	56	13.30
> 70	34	8.08
**Total**	**421**	**100.00**
University type		
Public	175	41.57
Private	246	58.43
**Total**	**421**	**100.00**
Plastic surgery specialty		
Yes	201	47.74
No	220	52.26
**Total**	**421**	**100.00**

The average age of the physicians accused of misconduct was 49.5 years. The youngest was 30 years old and the oldest, 73 years old. The majority (58.43%) were younger than 50 years at the time when the complaint was filed. Most of the defendants had received their medical degrees from private universities and were not specialists in plastic surgery.

Table 2 shows the breakdown of all the physicians without a specialty title in plastic surgery who were accused of medical misconduct, namely: physicians with a medical specialty other than physical surgery; and physicians bearing no record of any medical specialty.

**Table 2.** Distribution of the numbers of physicians without a specialty title in plastic surgery who were accused of medical misconduct, including physicians with a medical specialty other than plastic surgery and physicians with no record of any medical specialty, in numbers and percentages

Distribution of physicians without a specialty title in plastic surgery		
Other specialties	n	%
Anesthesiology	17	7.72
Angiology/vascular surgery	1	0.45
Oncology	1	0.45
General surgery	1	0.45
Vascular surgery	1	0.45
Internal medicine	1	0.45
Internal medicine − endocrinology and metabolic health	1	0.45
Dermatology	23	10.45
Endocrinology and metabolic health	1	0.45
Obstetrics and gynecology	5	2.27
Homeopathy − pediatrics	2	0.91
Family medicine and community health	1	0.45
Occupational health	3	1.36
Clinical nutrition	1	0.45
Ophthalmology	2	0.91
Orthopedics	5	2.27
Otorhinolaryngology	6	2.73
Pathology	1	0.45
No record of any specialty	147	66.88
**Total**	**220**	**100.00**

Among the 421 cases, 201 cases (47.74%) were brought against specialists in plastic surgery, 147 cases (34.91%) were brought against physicians who were not licensed in any specialty and 73 cases (17.34%) were brought against physicians who were licensed as medical specialists in fields other than plastic surgery.

### Classification of complaints

Table 3 lists the medical ethics code chapters (subject/topic) cited in the 421 cases. The most frequent subject/topic cited in these cases brought between 2008 and 2017 was violation of professional responsibility (medical malpractice, recklessness or negligence) (79.10%), followed by doctor-doctor relationships (complaints filed by fellow physicians) (52.49%) and then by professional confidentiality (34.67%).

**Table 3.** Distribution of medical ethics code chapters (subject/topic) cited in the 421 cases, in numbers (occurrences) and percentages

Breakdown of medical ethics code subjects/topics cited		
Ethics code subjects and topics	Occurrences	% (n = 421)
Professional responsibility	333	79.10
Human rights	95	22.56
Doctor-patient relationship	100	23.75
Doctor-doctor relationship	221	52.49
Professional remuneration	132	31.35
Professional confidentiality	146	34.67
Medical documents	141	33.49
Teaching and medical research	03	0.71
Medical advertising	137	32.54

The medical ethics code articles most often violated were articles 18 (35.82%), 51 (27.45%), 75 (20.22%) and 1 (16.51%). These are all deontological articles referring to professional responsibility, doctor-doctor relationships and professional confidentiality.

Among the cases filed over the study period, 233 cases (55.35%) had been adjudicated by the end of the year 2017, when data were collected for this review. The other 188 cases remained pending, awaiting the board’s ruling. Among the 233 adjudicated cases, 133 (57%) resulted in disciplinary sanction and 80 (34.5%) were ruled in the physician’s favor.

Table 4 shows the distribution of disciplinary sanctions taken in the 133 cases in which the physician was found guilty, with a breakdown according to sex, age, type of medical university and possession of a specialist title in plastic surgery.

**Table 4.** Breakdown of disciplinary sanctions taken in the 133 cases in which physicians were found guilty, with a breakdown by sex, age, type of medical university, and specialty title in plastic surgery status, in numbers and percentages

	Breakdown of disciplinary sanctions in 133 cases in which physicians were found guilty											
	Punishment A		Punishment B		Punishment C		Punishment D		Punishment E		Total	
	n	%	n	%	n	%	n	%	n	%	n	%
Sex												
Male	11	10.67	37	35.95	31	30.09	15	14.56	09	8.73	103	77.44
Female	3	10.00	10	33.33	13	43.33	04	13.34	0	0.00	30	22.56
**Total**	**14**	**10.52**	**47**	**35.33**	**44**	**33.08**	**19**	**14.28**	**09**	**6.79**	**133**	**100.00**
Age												
30-39	0	0.00	6	15.00	31	77.50	3	7.50	0	0.00	40	30.07
40-49	0	0.00	34	62.96	7	12.96	10	18.51	3	5.57	54	40.60
50-59	6	31.57	6	31.57	4	21.08	3	15.78	0	0.00	19	14.28
60-69	7	58.33	1	8.35	2	16.66	2	16.66	0	0.00	12	9.03
> 70	1	12.50	0	0.00	0	0.00	1	12.50	6	75.00	8	6.02
**Total**	**14**	**10.52**	**47**	**35.33**	**44**	**33.08**	**19**	**14.28**	**9**	**6.79**	**133**	**100.00**
University Type												
Public	3	6.00	17	34.00	17	34.00	6	12.00	07	14.00	50	37.59
Private	11	13.25	30	36.14	27	32.55	13	15.66	02	2.40	83	62.41
**Total**	**14**	**10.52**	**47**	**35.33**	**44**	**33.08**	**19**	**14.28**	**09**	**6.79**	**133**	**100.00**
Specialty title in plastic surgery												
Yes	7	11.66	26	43.35	23	38.33	4	6.66	0	0.00	60	45.11
No	7	9.58	21	28.76	21	28.76	15	20.54	9	12.36	73	54.89
**Total**	**14**	**10.52**	**47**	**35.33**	**44**	**33.08**	**19**	**14.28**	**9**	**6.79**	**133**	**100.00**

It was observed that male physicians were most often sanctioned with punishment B (35.95%), while female physicians were most often sanctioned with punishment C (43.33%). Regardless of the type of punishment, male physicians in general faced more punishment than female physicians. With regard to age group, physicians aged 30-39 years were most likely to be administered punishment C (77.50%), physicians aged 40-49 were most likely to be administered punishment B (62.96%), physicians aged 60-69 were most likely to be administered punishment A (58.33%) and physicians aged 70 and older were most likely to be administered punishment E (75.00%). Also, punishment C was most applied to doctors aged 30-39 (the youngest cohort), and punishment E was most applied to doctors aged 70 and over (the oldest cohort).

Regarding university type, physicians who studied at a private institution were more likely to face disciplinary sanction (62.41%) than were those who studied at public institutions (37.59%). Regardless of the type of institution, punishments B and C were administered most frequently.

Physicians without a medical specialty title in plastic surgery were more likely to face disciplinary sanction (54.89%) than were those who were specialists in plastic surgery. However, in looking more closely at the punishments applied to those with a specialty title in plastic surgery, punishments B and C (43.35% and 38.33% respectively) are seen to be most prevalent.

## DISCUSSION

### Sex

Out of the 273 physicians involved in the 421 cases analyzed here, 77.19% were male, a finding that is consistent with other studies.^16^^,^^18^ It was noted that complaints were brought against male physicians and that male physicians were found guilty of misconduct three times more often than was observed among female physicians.

Carvalho^19^ reported that complaints were filed less frequently against female physicians because they interacted more with patients, spent more time listening to them during examinations, saw fewer patients overall and treated patients with less serious conditions or complaints. In other words, women were perceived to be better at interacting, listening, talking and explaining, and they seemed to be more attentive as well. Despite the physician-physician relationship, this may explain the comparatively low number of complaints filed with regional medical boards against female physicians, compared with male physicians.

### Age

The average age of the physicians accused of misconduct was 49.5. The age range in which the greatest number of physicians was found guilty was the 40 to 49-year-old group (40.60%), while the oldest group (70+ years old) was the age group found guilty least often (6.02%).

These numbers referred to physicians who had been licensed for about 10 years and who were, therefore, in their most productive phase. At this time in their careers, they undertake a large volume of medical procedures, with high number of complex surgeries, while experiencing a time of greater financial demand relating to family and personal responsibilities.

The average age range of the physicians accused of misconduct, namely 40-49 years old, was consistent with the findings.^20^^,^^21^^,^^22^ This symmetry corroborates the results of this study.

### Medical specialty title in plastic surgery

This study shows that 220 of the cases in which complaints were filed (52.26%) were against physicians who were not medical specialists in plastic surgery. Brazilian law allows doctors to perform any procedure provided that they possess the requisite technical knowledge.^10^ Physicians licensed for other specialties are performing plastic surgeries for which they are not prepared, which increases patient risk. One study conducted by CREMESP in 2008 showed that 97% of the cases of mistakes (or misconducts) in plastic surgeries were filed against physicians who did not possess a specialist title in plastic surgery.^23^

In Brazil, given the shortage of residency places and given the personal need to begin working, it is common for physicians to begin practicing internal medicine and to informally practice plastic surgery. They may occasionally obtain a license in plastic surgery, following a review of their knowledge and experience by the medical association.^24^

It can be noted that a generalist profile is very common among physicians in small towns, while in big cities there is greater demand for specialized professionals.

Standardization of care, through better regulation and standards regarding medical specialization, is needed to ensure patient safety and to provide back-up for patients’ options in choosing a licensed plastic surgeon.^25^

### Medical ethics code chapters (subject/topic)

Violation of professional responsibility (medical malpractice, recklessness or negligence) was the most common complaint. This result is consistent with published data.^12^

The existence of complaints of negligence, medical malpractice or recklessness makes it clear that most mistakes or misconducts that occur in the practice of plastic surgery are due to physicians’ omissions or passiveness in relation to patients who should be receiving more attention and care. This is corroborated by the rate of cases found in physicians’ favor on account of a lack of evidence to back up the complaints.^21^

However, the general understanding is that it is legally permitted to practice medicine provided that the physician meets the legal and professional licensing requirements. It is illegal to practice medicine with any degree of recklessness, malpractice or negligence. When recklessness, medical malpractice or negligence occurs, Brazilian law calls for redress and punishment and establishes that damages should be awarded to the patient(s) harmed. In these situations, culpability is generally and usually attributed to the physician. Advances in medical science have led to greater medical responsibility, and, in this light, physicians are now liable for greater risks and for a greater number of possible accidents, which may be grounds for a discussion on culpability lying outside of physicians’ purview.^26^

Physicians filing complaints against their colleagues was the second most common plastic-surgery-related complaint filed (doctor-doctor relationship). The medical ethics code explicitly lists medical professionals’ rights and duties. Although it may seem unethical to file a complaint against a fellow physician, situations in which colleagues fail, through omission or commission, to honor the medical ethics code give rise to a duty among physicians to report their colleagues’ conduct to the regional medical board. The medical ethics code seeks to safeguard professional relationships.^27^

The problems that physicians may face in relation to their colleagues fall into two types: interpersonal matters like a lack of communication, cooperation or harmony; and professional matters like anti-ethical self-promotion or publicizing of “foolproof” methods and innovative and exclusive techniques, etc. Such violations consequently lead to a breakdown of trust among physicians and weaken professional relationships, as stated in the medical ethics code of 2009. It is therefore important that physicians do file complaints before the regional medical board so that it can investigate any violations of the code. Since these complaints are considered to be administrative cases, physicians need not be wary of filing complaints; by bringing cases to the board’s attention, they are fulfilling their civic duty.

In relation to professional confidentiality, a distinction is made between ethics and morality. Confidentiality has always been the moral obligation of professionals working in the field of medicine. Morality, like confidentiality, involves principles that guide a given behavior, while ethics consists of philosophical discussions for critical evaluation of morality and professional confidentiality. Therefore, ethics is a treatise within morality, dealing with values on scales from good to bad and from right to wrong. It needs to be noted that, despite the existence of a moral compass in the medical profession that is codified through rules to guide proper behavior, some physicians do not follow this and fail to respect confidentiality between doctors, patients and family information.

### Medical ethics code articles most commonly violated

This study showed that the four most commonly violated ethics code articles among those in effect when the complaints were filed were 18, 51, 75 and 1. Article 18 (“Disobeying or disrespecting Brazilian Medical Association (CFM) or regional medical council rulings or appellate decisions”) was cited most often in a previous study on complaints and cases against physicians who were found guilty and received punishment; those findings are corroborated by the results from the present study.^28^ Article 51 (“Engaging in unfair competition with another physician”) primarily relates to self-promotion. Article 75 (“Making reference to identifiable clinical cases or exposing patients or their profiles in medical advertisements, medical publications or the media in general without patient consent”) is most often found in marketing and advertising involving pre and postoperative patient photos. Lastly, article 1 (“Causing harm to the patient, through omission or commission, characterized by medical malpractice, recklessness or negligence”) is referred to collectively in the present paper as “professional misconduct”.^29^

Although medical advertising was not the subject of the majority of the complaints filed during the period of the present study, all four of the aforementioned articles involve medical advertising to some degree, as explained in a previous study.^30^ In plastic surgery, medical advertising strongly influences the mistake or misconduct identified. Profiting from the profession through marketing of medicine is considered to be unethical behavior. In this vein, physicians are not allowed to be party to any commercials or advertisements that in any way promote or profit from the profession. Regardless of the specialty, physicians should not guarantee results or treatment. Physicians must clearly inform patients of the benefits and risks of a given procedure. Advertising in which physicians publicize simple and quick treatment that is 100% effective is an invitation for lawsuits to hold physicians to the purported results. Promising results put the physician in a delicate situation, since complications may arise during treatment. Medical information should include what is scientifically correct and accepted as good medical practice, and medical professionals should base their practice on the laws that are in effect.

### Disciplinary sanctions

Our review of the disciplinary sanctions taken showed that punishment B (confidential reprimand - classified notice) was the punishment most often administered. This finding speaks to one of CREMESP’s maxims, namely that professional-misconduct cases are meant to serve as a means for educating physicians. There is generally no reason to put the ruling or sanctions imposed into the public record, except in cases in which the situation is grave, or in cases of repeated offense and cases in which there is imminent indisputable risk or harm to others. These findings are similar to previous findings regarding the prevalence of confidential disciplinary sanctions.^16^^-^^31^

The data shows that punishment C was applied most to doctors aged 30-39 (the youngest group), and punishment E was applied most to doctors aged 70 and over (the oldest group).

It is not surprising that a punishment of public record was applied most to doctors in the 30-39-year-old cohort. Physician behavior at this age perfectly explains this phenomenon. The heavy workload facing the youngest group means that there is greater likelihood of facing a complaint. Moreover, self-confidence and possible consequent carelessness explain complaints filed against the oldest group. This supports similar findings that negligence was the complaint most often cited, reported in some previous studies.^21^^,^^22^

Physicians without a medical specialty title in plastic surgery were more likely to face disciplinary sanction (54.89%) than those who were specialists in plastic surgery. However, looking more closely at the punishments applied to those with a specialty in plastic surgery, punishments B and C (43.35% and 38.33% respectively) were seen to be most prevalent. Greater severity of disciplinary sanctions for certain violations shows the existence of concern regarding where the field of medicine is heading and, primarily, concern regarding safeguarding society from medical malpractice, which is the *raison d’être* of the regional medical boards.

## CONCLUSION

The classification of physicians against whom plastic surgery medical misconduct cases were filed before the São Paulo State Medical Board (CREMESP) that was presented in this study was similar to what has been shown elsewhere in the medical literature. Physicians of all medical specialties, including plastic surgeons, are aware that medical practice nowadays is replete with many ethical assumptions and bioethical dilemmas stemming from new technologies and procedures. This demands deeper ethical reflection before making medical decisions. Medical misconduct due to negligence has become more rigorously punished on ethical grounds, both in Brazil and internationally.
